# SH2B3, Transcribed by STAT1, Promotes Glioblastoma Progression Through Transducing IL-6/gp130 Signaling to Activate STAT3 Signaling

**DOI:** 10.3389/fcell.2021.606527

**Published:** 2021-04-13

**Authors:** Shan Cai, Jian-xiang Lu, Yan-pei Wang, Chao-jia Shi, Tian Yuan, Xiang-peng Wang

**Affiliations:** Department of Neurosurgery, First Affiliated Hospital of Kunming Medical University, Kunming, China

**Keywords:** SH2B3, STAT1, STAT3, glioblastoma, glioblastoma stem cell

## Abstract

Glioblastoma (GBM) is the most common and aggressive brain tumor in adults. The aberrant activation of STAT3 commonly occurs in GBM and is a key player in GBM tumorigenesis. Yet, the aberrant activation of STAT3 signaling is not fully understood. Here, we report that SH2B adaptor protein 3 (SH2B3) is highly expressed in GBM and preferentially expressed in GBM stem cells (GSCs). Moreover, SH2B3 high expression predicts worse survival of GBM patients. Targeting SH2B3 considerably impairs GBM cell proliferation, migration, and GSCs’ self-renewal *in vitro* as well as xenograft tumors growth *in vivo*. Additionally, we provide evidence suggesting that STAT1 directly binds to the promoter of SH2B3 and activates SH2B3 expression in the transcriptional level. Functionally, SH2B3 facilitates GBM progression *via* physically interacting with gp130 and acting as an adaptor protein to transduce IL-6/gp130/STAT3 signaling. Together, our work firstly uncovers that the STAT1/SH2B3/gp130/STAT3 signaling axis plays critical roles in promoting GBM progression and provides insight into new prognosis marker and therapeutic target in GBM.

## Introduction

Glioblastoma (WHO grade IV) is the most common and malignant brain tumor in adults (Brennan et al., 2014). Although treated with radical regimens, including surgical resection, and radiotherapy with concomitant or adjuvant temozolomide (TMZ) chemotherapy, the median survival of GBM patients is only 15 months (Stupp et al., 2005), and the 5-year survival rate is only 5.5% (Ostrom et al., 2019). Glioma stem cells (GSCs) are subpopulation glioma cells that reside at the hierarchical apex, which was considered to account for glioma initiation, malignant progression, therapeutic resistance, and tumor recurrence (Bao et al., 2006a, b; Chen et al., 2012). Thus, strategies to specifically deplete GSCs may be translated into new promising therapies, which are urgently needed. TMZ is an oral alkylating agent that is commonly used to treat GBM and astrocytoma (Batchelor, 2000; Yung et al., 2005; Van Meir et al., 2010). However, at least 50% of GBM patients who received TMZ treatment eventually develop resistance to TMZ, highlighting an urgent need to understand the molecular mechanism underlying TMZ resistance of GBM. Numerous studies reported that various genes play important roles in promoting TMZ resistance of GBM, and targeting those key regulators sensitizes GBM to TMZ (Kohsaka et al., 2012; Li et al., 2017; Liao et al., 2017; Kim et al., 2018). Thus, elaborating the underlying mechanisms of TMZ resistance in GBM patients may provide new insights into GBM patients’ therapy.

The aberrant activation of STAT3 is essential for maintaining the tumorigenic potential of GSCs (Guryanova et al., 2011). STAT3 activation is a key signaling event that is both required for malignant and normal cells’ physiological functions (Yu et al., 2009; Guryanova et al., 2011). The canonical STAT3 signaling is stimulated by the interleukin-6 (IL-6) cytokine family, followed by the dimerization of the IL-6 receptor glycoprotein 130 (gp130) and then phosphorylated and activated by Janus family kinases (JAKs) (Yu et al., 2009). Activated JAKs phosphorylate and activate STAT3 *via* inducing its nuclear translocation to promote transcription of target genes (Chen et al., 2015). Thus, identifying the underlying molecular mechanisms of the aberrant activation of STAT3 in GSCs may accelerate the progression of therapeutics by selectively deleting GSCs.

SRC homology domain is an evolutionarily conserved domain in the non-catalytic part of the receptor tyrosine kinases, which was well established and played crucial roles in signaling transduction and various biological processes (Lin et al., 2020). SH2B3 was first identified in T cells and originally characterized as LNK. Subsequently, SH2B3 was identified as a signal transduction regulator that was implicated in modulating various cytokine signaling cascades (Blass et al., 2016). As a member of the family of SH2B adaptor proteins, SH2B3 harbors several functional domains, including the SRC homology 2 (SH2) domain that is essential for its binding with target proteins and the pleckstrin homology (PH) domain that recognizes phosphoinositides and is translocated to the cell membrane. Recent advancement has shown that the dysregulation of SH2B3 was associated with multiple diseases, including atherosclerosis and thrombosis (Wang et al., 2016), hypertension (Rudemiller et al., 2015), and cancer (Devalliere and Charreau, 2011; Maslah et al., 2017). SH2B3 was shown to bind to and negatively regulate several crucial signaling pathways, such as Janus kinases and other tyrosine kinases regulating cytokine receptors. SH2B3 was extensively studied in malignant hematopoietic diseases and functions as a tumor suppressor gene in leukemogenesis (Gery and Koeffler, 2013; Perez-Garcia et al., 2013; Cheng et al., 2016; Sinclair et al., 2019). In contrast to hematologic malignancies, SH2B3 was reported to activate the JAK/STAT3 and ERK1/2 pathways to promote the progression of triple-negative breast cancer (Lv et al., 2020) and act as a positive signal transduction regulator in ovarian cancers (Ding et al., 2015). Another study identified SH2B3 as a colon cancer susceptibility gene (Raskin et al., 2017). However, the biological functions of SH2B3 in GBM initiation and development remain elusive. Based on this background, we wanted to investigate the functional roles of SH2B3 in GBM tumorigenesis.

## Materials and Methods

### Cell Lines

Human glioblastoma (GBM) cell lines (U87, U251, and T98G) used in this study were all purchased from ATCC (Manassas, United States) and then maintained in Dulbecco’s Modified Eagle’s Medium (DMEM) (Life Technologies). The DMEM culture medium was supplemented with 10% fetal bovine serum (FBS) and 1% antibiotics. The cells were all cultured under the condition of 95% O_2_, 5% CO_2_, and 37°C.

### Lentivirus Production and Transduction

Short hairpin RNA (shRNA) directly targets human SH2B adaptor protein 3 (SH2B3), or scrambled oligonucleotides were ligated into the pLKO.1 vector. HEK293T cells were used to co-transfect with psPAX2 and pMD2.G and the indicated lentiviral vectors using Lipofectamine 3000 (Life Technologies Corporation, United States). After 48 h of transfection, lentiviral particles in the supernatant were collected and filtered by 0.22 μm filter membrane. Indicated cells were then infected with lentivirus, and stable cell lines were selected with 1 μg/ml puromycin for 2 weeks. The sequences of shRNA oligonucleotides are listed in Table 1.

**TABLE 1:** Primer sequences used in this study.

**Sequences of PCR primers used in this study**	
**Gene**	**Sequence**
β-Actin-F	5′-GGGAAATCGTGCGTGACATTAAG-3′
β-Actin-R	5′-TGTGTTGGCGTACAGGTCTTTG-3′
SH2B3-F	5′-TTGAGATGCCTGACAACCTTTAC-3′
SH2B3-R	5′-GCTCTAGGGCTGAGGGAATATG-3′
Gli1-F	5′-AGCGTGAGCCTGAATCTGTG-3′
Gli1-R	5′-CAGCATGTACTGGGCTTTGAA-3′
Gli3-F	5′-GAAGTGCTCCACTCGAACAGA-3′
Gli3-R	5′-GTGGCTGCATAGTGATTGCG-3′
CCND1-F	5′-GCTGCGAAGTGGAAACCATC-3′
CCND1-R	5′-CCTCCTTCTGCACACATTTGAA-3′
TCF4-F	5′-CAAGCACTGCCGACTACAATA-3′
TCF4-R	5′-CCAGGCTGATTCATCCCACTG-3′
HEY1-F	5′-AGGGCTACAGCCTGTGCCCAG-3′
HEY1-R	5′-AGGGCTACAGCCTGTGCCCAG-3′
HEY3-F	5′-GTTCGGCTCTAGGTTCCATGT-3′
HEY3-R	5′-CGTCGGCGCTTCTCAATTATTC-3′
STAT3-F	5′-CAGCAGCTTGACACACGGTA-3′
STAT3-R	5′-AAACACCAAAGTGGCATGTGA-3′
SOCS3-F	5′-CCTGCGCCTCAAGACCTTC-3′
SOCS3-R	5′-GTCACTGCGCTCCAGTAGAA-3′
**shRNA**	**Sequence**
sh-STAT1	5′-GAACCAACTGTCTTGGCATTC-3′
sh-SH2B3	5′-GCTCAGAAGGACATGGGATTC-3′
sh-gp130	5′-TACAGAACTGTACAACTCGTG-3′
**Sequence for Ch-IP primers**	
Potential binding site 1-F	5′-CCTCACAACCCTCCATGGCAC-3′
Potential binding site 1-R	5′-CAAAGGAACAGCATGTGCAAA-3′
Potential binding site 2-F	5′-TGGAGCCAAGCCTCGAGCTAA-3′
Potential binding site 2-R	5′-GGGGCCACCTCGAGTCTTCCC-3′

### Immunohistochemistry

Immunohistochemistry (IHC) analysis of mouse xenograft tumor tissues was performed in accordance with the standard protocol. Briefly, paraffin-embedded tissues were cut into 4-μm thick sections. The sections were followed by antigen retrieval after being de-paraffinized and rehydrated through a descending alcohol series. The sections were incubated with antibodies against Ki67 and p-STAT3 overnight at 4°C after blocking with 1% bovine serum albumin (BSA) for 25 min at room temperature. Then, the sections were incubated with secondary antibody conjugates with horseradish peroxidase (HRP) for 30 min at 37°C and examined by using diaminobenzidine detection.

### Colony Formation Assays

U251 and U87 expressing with or without SH2B3 shRNA cells were seeded to 6-well plates at a density of 3,000 cells per well. The cells were cultured for 2 weeks to form the colonies. The colonies were stained with 0.05% crystal violet (HBK Pharmaceutical Technology Co., China) for 30 min. After carefully washing with phosphate-buffered saline (PBS), the colonies were imaged, and colonies larger than 50 cells were counted. The experiment was performed in triplicate.

### BrdU Incorporation Assay

Single cells were seeded to 48-well plates at a density of 40,000 cells per well. After culturing for 24 h, the cells were treated with 100 μM 5-bromo-2′-deoxyuridine (BrdU). Then, the cells were fixed and incubated with primary antibody against BrdU antibody (5292; Cell Signaling Technology). At least five random fields were examined for BrdU positive cells.

### Dual-Luciferase Assay

Indicated cells were seeded into 12-well plates and transfected with pGL4 luciferase vector (Addgene) containing with or without the promoter of SH2B3. Transfection efficiency was quantified by co-transfection with Renilla luciferase reporter. The activities of firefly luciferase and Renilla luciferase in each well were examined by a dual-luciferase reporter assay system (Promega). The ratios between the luciferase reporter and Renilla control were determined 48 h after transfection. The relative luciferase activity of the SH2B3 promoter–luciferase activities was further normalized to that in the cells transfected with the firefly luciferase vector control under the same treating conditions. The SH2B3 promoter activity was also normalized by co-transfection with the Renilla luciferase reporter.

### Western Blot

Glioblastoma cells were washed in PBS and lysed with ice-cold RIPA buffer (Beyotime, P0013B, China) containing protease inhibitor (Beyotime, P1010, China). The same quality protein was subjected to sodium dodecyl sulfate-polyacrylamide gel electrophoresis (SDS-PAGE) and transferred to a polyvinylidene difluoride membrane. After blocking with 5% milk, membranes were immunoblotted with specific antibodies according to standard protocols. Antibodies used in this study were shown below. SH2B3 was purchased from Abcam (ab154848). STAT3 (12640), p-STAT3 (9145), STAT1 (14994), and Ki67 (9449) were all purchased from Cell Signaling Technology.

### Chromatin Immunoprecipitation Assay

Chromatin immunoprecipitation (Ch-IP) assay was performed in both U87 and U251 cell lines using a Ch-IP kit (Millipore, United States) according to the manufacturer’s instructions. Briefly, after cross-linking, the cells were lysed and sonicated (15 s for five times). Sonicated lysates were centrifuged at 15,000 rpm at 4°C for 15 min, and the cleared chromatin (100 μg) was immunoprecipitated with 5 μg of anti-STAT1 antibody and incubated overnight at 4°C with rotation. Quantitative PCR was performed for the detection of the bind site. The primer sequence used in this assay is listed in Table 1.

### Quantitative RT-PCR

Total RNA was extracted with the RNeasy kit (QIAGEN) and reversely transcribed to cDNA with PrimeScript RT Master Mix (Takara Bio Inc.). Real-time PCR was performed on a cycler (Applied Biosystems) using SYBR green master mix (Takara Bio Inc.). The sequences of primers used in this study are listed in Table 1.

### Apoptotic Analysis by Flow Cytometry

Annexin V–FITC and propidium iodide (PI) staining was performed for flow cytometry according to the manufacturer’s guidelines (BD). In brief, GBM cells were transduced with SH2B3 shRNA or control shRNA treated with 50 μM for 48 h. Then, all cells were collected and resuspended with 100 μl of binding buffer and incubated with 5 μl PI and 5 μl annexin V–FITC staining for 30 min in the dark at room temperature. Flow cytometric analysis was immediately performed using a flow cytometer (LSR II; BD).

### Cell Proliferation Assay

U251 and U87 expressing with or without SH2B3 shRNA cells were seeded to 12-well plates at a density of 20,000 cells per well. The number of cells was counted at indicated times and normalized on day 0.

### Transwell Assay

Cell migration ability was assessed by using 8-μm transwell inserts (Corning, United States). Briefly, a total of 3,000 U251 and U87 expressing with or without SH2B3 shRNA cells were resuspended in 100 μl serum-free culture medium and seeded into the upper chambers, and the lower compartment was filled with 600 μl medium containing 10% FBS. After culturing for 24 h at 37°C, non-migrating cells on the upper chambers were removed by using a cotton swab, and the cells adhering to the bottom side of inserts were fixed with 4% paraformaldehyde (PFA) for 25 min, followed by staining with 0.1% crystal violet for 60 min. After rinsing with PBS, migrating cells were imaged by a microscope and counted. The experiments were performed in triplicate.

### Immunofluorescence Staining

Briefly, 4% PFA was used to fix human surgical glioma specimens overnight. Samples were blocked with 10% normal donkey serum supplemented with 0.2% Triton X-100 in PBS for 30 min at room temperature and then incubated with primary antibodies overnight at 4°C, followed by incubation with appropriate secondary fluorescently labeled antibodies (Invitrogen) for 2 h at room temperature. Nuclei were counterstained with Hoechst. Two GBM patients’ clinical specimens were used in this study.

### Co-immunoprecipitation

The cells were harvested and lysed with RIPA buffer containing protease inhibitors for 30 min at 4°C. Cell lysates were pre-cleared by protein A/G beads (Santa Cruz Biotechnology) for 1 h incubation at 4°C. Pre-cleared protein A/G beads were removed. Then, primary antibody was added for overnight incubation at 4°C. New protein A/G beads were added for 2 h incubation. Then, beads were collected by washing with ice-cold PBS for four times. Finally, the bound protein was eluted by boiling for 10 min, subjected to SDS-PAGE, and detected by immunoblotting. SH2B3 (Abcam ab154848, 1:100), gp130 (sc-655, 1:100, Santa Cruz).

### Wound Healing Assay

Indicated cells were seeded into six-well plates at a density of 600,000 cells per well, and the scratch was generated using a 200 μl pipette tip when the cells were grown to 80% confluence. The wound closure was imaged under a microscope at 0 and 48 h after being maintained in serum-free medium.

### Tumor Sphere Formation and *in vitro* Limiting Dilution Assay

For tumor sphere formation assay, single-cell suspensions were seeded in 6-well ultra-low attachment plates (Corning Inc., Corning, United States) at a density of 10,000 cells per well using serum-free DMEM/F12 (Hyclone) containing 20 ng/ml of basic fibroblast growth factor (Miltenyi Biotec), 20 ng/ml of epidermal growth factor (Miltenyi Biotec, Auburn, United States), and 2 mM L-glutamine (Mediatech Inc.). After culturing for 14 days, the number of formed tumor spheres was counted and pictured using a microscope. For *in vitro* limiting dilution assay, dissociated single U87 and U251 cells were seeded in ultra-low attachment 96-well plates at the density of 5, 10, 20, 50, 100, or 200 cells per well. After 14 days of culture without serum, spheres formatted were counted in each well, and sphere formation frequency was calculated using online *in vitro* limiting dilution analysis^1^.

### *In vivo* Tumor Growth Model

Xenograft tumor model was created through subcutaneous injection with 1 × 10^6^ U87 cells expressed with control shRNA or SH2B3 shRNA to BALB/c nude mice. Tumor volume was recorded twice a week. Six weeks later, mice were sacrificed, and tumors derived from indicated cells were collected, weighed, and pictured. All animal studies were approved by the Animal Ethics Committee of Kunming Medical University.

### Bioinformatic Analysis

To comprehensively understand the expression pattern of SH2B3 and its prognostic implications in gliomas, transcriptome sequencing and their corresponding clinical data of TCGA, CGGA, and Rembrandt data sets were downloaded from http://gliovis.bioinfo.cnio.es/.

### Statistical Analyses

Each experiment was repeated at least three times. Unless otherwise noted, data are presented as mean ± SD and analyzed by Student’s *t*-test (unpaired, two-tailed) using GraphPad Prism 6.0. Survival analysis was evaluated using the Kaplan–Meier method and assessed using the log-rank test. *p* < 0.05 was considered statistically significant.

## Results

### Expression and Clinical Implications of SH2B3

**FIGURE 1:**
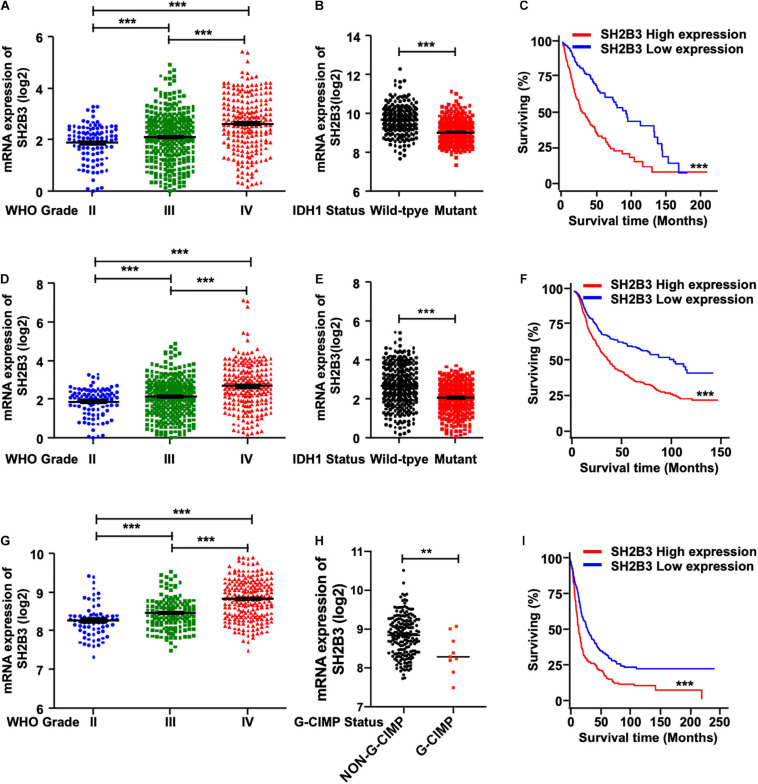
SH2B3 expression is elevated in glioblastoma. **(A)** SH2B3 expression in different grades of gliomas in the TCGA data set. **(B)** SH2B3 expression in the IDH1 WT and mutant groups was analyzed in the TCGA data set. **(C)** The correlation of SH2B3 expression and its corresponding glioma patient’s survival in the TCGA data set were analyzed. Survival analysis was evaluated using the Kaplan–Meier method and assessed using the log-rank test. **(D)** SH2B3 expression in different grades of gliomas in the CGGA data set. **(E)** SH2B3 expression in the IDH1 WT and mutant groups was analyzed in the CGGA data set. **(F)** The correlation of SH2B3 expression and its corresponding glioma patient’s survival in the CGGA data set were analyzed. Survival analysis was evaluated using the Kaplan–Meier method and assessed using the log-rank test. **(G)** SH2B3 expression in different grade gliomas in Rembrandt data set. **(H)** SH2B3 expression in the G-CIMP and non-G-CIMP groups was analyzed in Rembrandt data set. **(I)** The correlation of SH2B3 expression and its corresponding glioma patient’s survival in Rembrandt data set were analyzed. Survival analysis was evaluated using the Kaplan–Meier method and assessed using the log-rank test. *, **, and *** indicate *p* < 0.05, *p* < 0.01, and *p* < 0.0001, respectively.

To elucidate the potential clinical significance of SH2B3 in glioma, we first examined the message RNA expression pattern of SH2B3 in TCGA data set and found that SH2B3 was dramatically and highly expressed in GBM (WHO grade IV) compared with glioma (WHO grades II and III) (Figure 1A). Isocitrate dehydrogenase 1 (IDH1) mutational status has been well recognized to be an important prognosis biomarker, and patients with IDH1 mutant gliomas commonly have a favorable prognosis compared with patients with wild-type (WT) IDH1 tumors (Noushmehr et al., 2010; Turcan et al., 2012; Eckel-Passow et al., 2015). By comparing the expression of SH2B3 in the IDH1 WT and mutant groups, we observed that SH2B3 is significantly highly expressed in IDH1 WT gliomas (Figure 1B). Additionally, an elevated expression of SH2B3 predicts poor survival of glioma patients (Figure 1C), implying that SH2B3 has a potential to serve as a prognostic marker of glioma. Consistently, we observed almost exact patterns in CGGA (Figures 1D–F) and Rembrandt (Figures 1G–I) data sets. Notably, Glioma-CpG island methylator phenotype (G-CIMP) status is another potent prognostic biomarker, and patients with G-CIMP frequently have favorable prognosis (Baysan et al., 2012). By comparing the expression of SH2B3 in the G-CIMP and non-G-CIMP groups, we found that SH2B3 was highly expressed in the non-G-CIMP group (Figure 1H), which is consistent with the above findings. Together, these data strongly suggest that SH2B3 has important clinical significance for GBM patients’ prognosis.

### SH2B3 Is Preferentially Expressed in GSCs and Is a Direct Transcriptional Target Gene of STAT1

**FIGURE 2:**
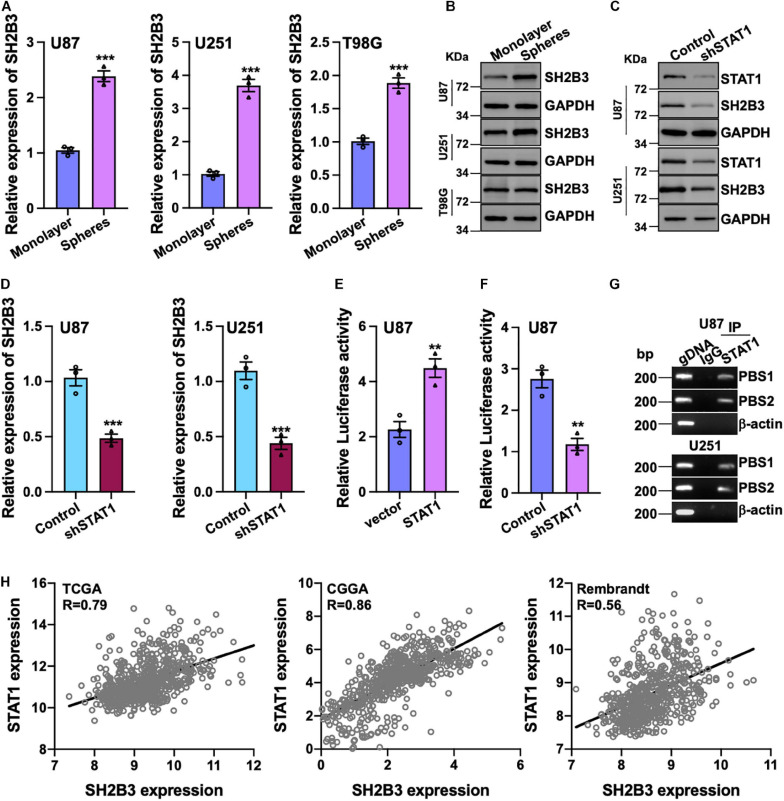
SH2B3 is a direct transcriptional target gene of STAT1. **(A,B)** Both mRNA **(A)** and protein **(B)** levels of SH2B3 were increased in tumor spheres derived from indicated cells compared with their corresponding monolayer cells. **(C,D)** Silencing of STAT1 decreased both SH2B3 protein **(C)** and mRNA **(D)** levels. **(E)** Luciferase activity of vector containing SH2B3 promoter in response to depletion of STAT1. **(F)** Luciferase activity of vector containing SH2B3 promoter in response to overexpression of STAT1. **(G)** STAT1 directly binds to SH2B3 promoter determined by Ch-IP assay. PBS means potential binding site. BP means base pair. β-Actin promoter was used as a negative control. gDNA (genomic DNA) was used as a positive control. **(H)** STAT1 mRNA expression was positively correlated with SH2B3 mRNA expression in the indicated data set. *, **, and *** indicate *p* < 0.05, *p* < 0.01, and *p* < 0.0001, respectively.

To further investigate the potential biological function of SH2B3 in GSCs’ self-renewal, we performed tumor spheres formation assay, which is a widely used assay to assess tumor stem cell’s self-renewal ability, in various GBM cell lines. We observed that tumor spheres derived from U87, U251, and T98G cell lines were expressed higher in SH2B3 than in their corresponding monolayer cells at both protein and mRNA levels (Figures 2A,B), implying that SH2B3 may play crucial roles in GSCs’ self-renewal.

To further explore the underlying mechanism of the elevated expression of SH2B3 in GBM and GSCs, we examined the potential transcription factors that may bind to the promoter of SH2B3 in online database gene cards^2^. As SH2B3 was reported to inhibit STAT1 signaling in melanoma (Bao et al., 2006b), to our surprise, the result showed that STAT1 may be the transcription factor that binds to the promoter of SH2B3 and STAT1 is dramatically highly expressed in GBM and promotes GBM cell proliferation and migration as well as GSCs’ self-renewal (Hua et al., 2019; Zhao et al., 2020). To test this hypothesis, we established cell lines that stably knock down STAT1 in U87 and U251 cells. As shown in Figures 2C,D, both mRNA and protein levels of SH2B3 were considerably decreased following silencing of STAT1, implying that SH2B3 may be a transcriptional target gene of STAT1. We next explored that STAT1 activates SH2B3 expression whether through directly binding to SH2B3 promoter. DNA fragments of SH2B3 promoter were inserted into a firefly luciferase reporter plasmid and introduced into U87 and U251 cells expressing with or without STAT1 overexpressed plasmid. As expected, luciferase expression from the forced expression of STAT1 cells, but not the empty vector cells, was markedly induced (Figure 2E). This STAT1-induced luciferase expression, however, was greatly inhibited by knockdown of STAT1 (Figure 2F). Ch-IP assay was performed to further confirm this observation. As shown in Figure 2G, STAT1 directly binds to SH2B3 promoter and activates SH2B3 expression. Consistently, we found that STAT1 mRNA expression was significantly positively correlated with SH2B3 mRNA in the three above-mentioned data sets (Figure 2H). Taken together, these data strongly support that SH2B3 was significantly increased in GSCs and STAT1 binds to the promoter of SH2B3, thereby activating SH2B3 in the transcriptional level.

### SH2B3 Is Required for GBM Cell Proliferation and Migration

**FIGURE 3:**
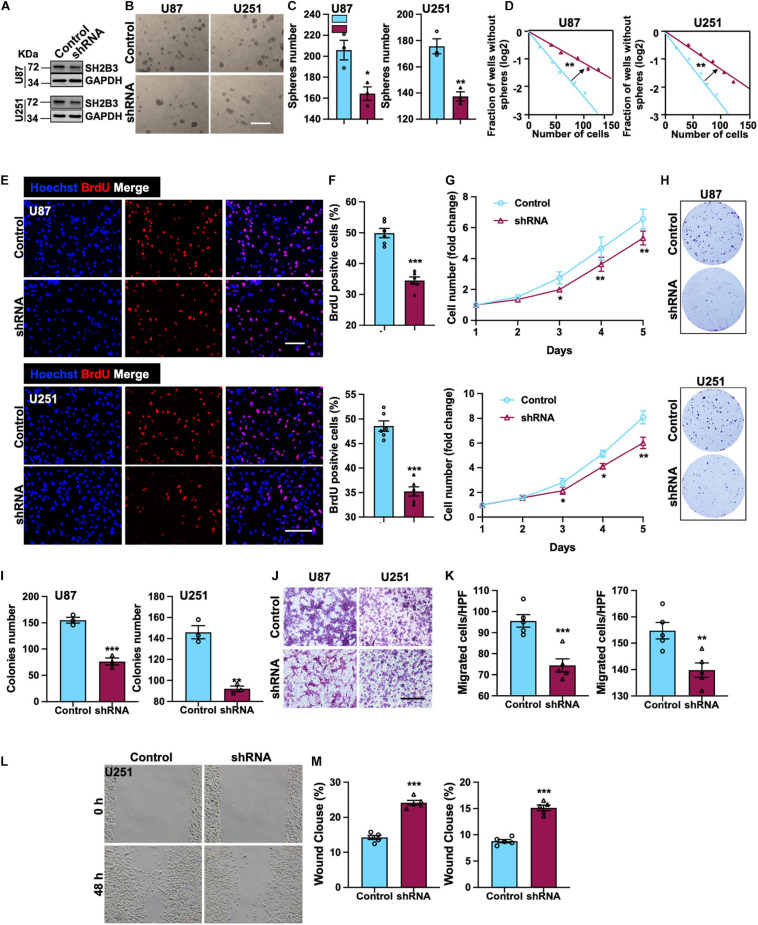
SH2B3 is required for GSCs’ self-renewal and GBM cell proliferation and migration. **(A)** Expression pattern of SH2B3 in indicated control or shSH2B3 cells. **(B)** Tumor spheres formation ability was inhibited by depleting SH2B3, and representative images of tumor spheres from indicated cells were shown. Scale bar = 500 μm. **(C)** Quantification data for panel **(B)**. **(D)** Indicated derived cell tumor spheres formation ability was repressed by depleting SH2B3 as determined by *in vitro* limiting dilution assay, and the quantification data were shown. **(E)** Immunofluorescence staining of BrdU positive cells in indicated cells and representative images were shown. Red dye represents proliferating cells. Scale bar = 100 μm. **(F)** Quantification data for panel **(E)**. **(G)** The cell proliferation rate of indicated cells expressing with or without SH2B3 shRNA. **(H)** Colony formation ability was inhibited by depleting SH2B3, and representative images of colonies derived from indicated cells were shown. **(I)** Quantification data for panel **(H)**. **(J)** Migration ability of indicated cells expressing with or without SH2B3 shRNA and representative images of migrated cells were shown. Scale bar = 100 μm. **(K)** Quantification data for panel **(J)**. **(L)** Indicated cells migration ability was inhibited by silencing of SH2B3 as assessed by wound healing assay, and representative images were shown. **(M)** Quantification data were shown. Control or shRNA means indicated cells expressing control shRNA or SH2B3 shRNA, respectively. *, **, and *** indicate *p* < 0.05, *p* < 0.01, and *p* < 0.0001, respectively.

To elucidate the potential functional roles of SH2B3 in glioma initiation and development, we established cell lines that stably knock down SH2B3 in U251 and U87 cell lines (Figure 3A), which were frequently used in GBM study. Tumor spheres formation assay was performed, and we found that tumor spheres formation ability was dramatically repressed following silencing of SH2B3 (Figures 3B,C), implying that SH2B3 is essential for GBM cell growth. *In vitro* limiting dilution assay data further validate this observation. As shown in Figure 3D, silencing of SH2B3 markedly suppressed GBM cell tumor sphere formation ability. Consistently, we observed that SH2B3 depletion cells have less BrdU positive cells as determined by BrdU incorporation assay in U251 and U87 cells (Figures 3E,F). Cell proliferation rate was significantly inhibited by depleting SH2B3 as determined by cell proliferation and colony formation assay (Figures 3G–I). Next, we further explore the biological function of SH2B3 in GBM infiltrative characteristic, and we observed that cell migration ability was significantly inhibited by SH2B3 knockdown as determined by transwell and wound healing assay (Figures 3J–M). Collectively, these results reveal that silencing of SH2B3 significantly impairs GBM cell proliferation and migration *in vitro*.

### Combination Silencing of SH2B3 With TMZ-Induced Cell Death *in vitro*

**FIGURE 4:**
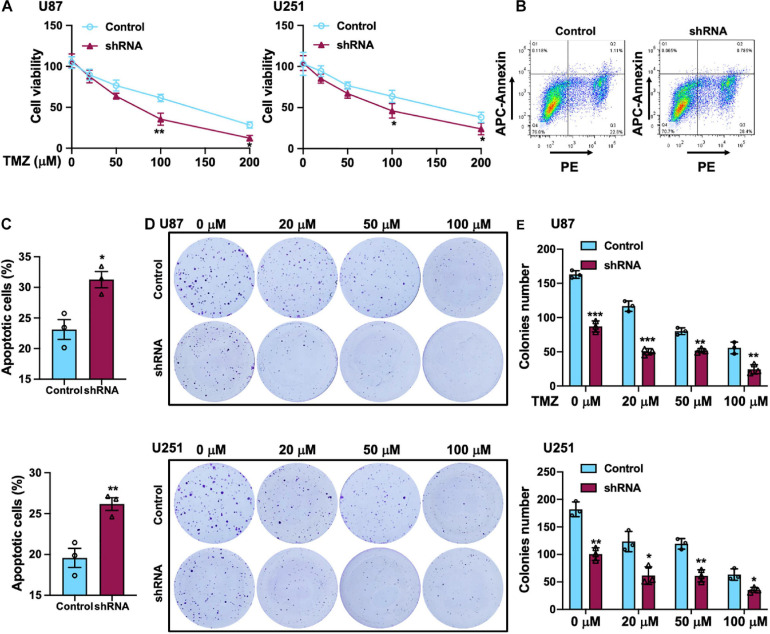
SH2B3 promotes TMZ-induced cellular apoptosis *in vitro*. **(A)** U87 (left panel) and U251 (right panel) cells expressing with or without SH2B3 shRNA were treated with indicated concentrations of TMZ, and cell viability was determined by sulforhodamine B (SRB) assay. **(B)** U87 cells expressing with or without SH2B3 shRNA were treated with 50 μM TMZ for 48 h, and living cells were assessed by fluorescence-activated cell sorting (FACS), and representative images were shown. **(C)** Quantification data of U87 (**B**, up panel) and U251 cells (below panel). **(D)** Representative images of colony derived from indicated cells were treated with indicated concentrations of TMZ. **(E)** Quantification data for panel **(D)**. Control or shRNA means indicated cells expressing control shRNA or SH2B3 shRNA, respectively. *, **, and *** indicate *p* < 0.05, *p* < 0.01, and *p* < 0.0001, respectively.

Temozolomide is an oral alkylating agent that is routinely used for GBM patients’ clinical treatment. However, at least 50% of GBM patients eventually do not appear to response to TMZ. Silencing of SH2B3 amplified TMZ-induced GBM cell repression of U251 and U87 cells proliferation and led to a marked increase of TMZ IC_50_ (Figure 4A). In addition, 50 μM TMZ treatment of U87 and U251 cells expressing with or without SH2B3 shRNA and SH2B3 silencing cells is significantly sensitive to TMZ treatments (Figures 4B,C). Disrupting SH2B3 in U87 and U251 cells augments inhibition colony formation ability after different concentrations of TMZ treatment (Figures 4D,E).

### Knockdown of SH2B3 Augments TMZ Sensitivity *in vivo*

**FIGURE 5:**
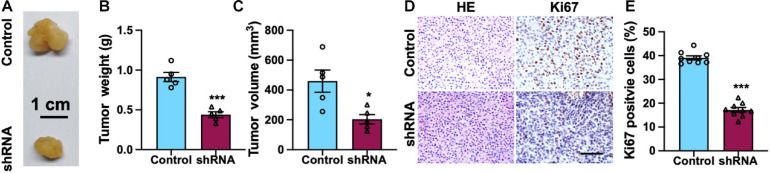
Silencing of SH2B3 augments TMZ-induced suppression of mouse xenograft tumors growth. **(A)** Representative images of tumors derived from U87 control or shRNA cells. Scale bar = 1 cm. **(B)** Weight of indicated tumors. **(C)** The volume of indicated tumors. **(D)** Immunohistochemistry staining with tumors derived from U87 control or SH2B3 shRNA cells using Ki67 antibody and representative images were shown. Scale bar = 100 μm. **(E)** Quantification data for panel **(D)**. Control or shRNA means indicated tumors derived from cells expressing control shRNA or SH2B3 shRNA, respectively. *, **, and *** indicate *p* < 0.05, *p* < 0.01, and *p* < 0.0001, respectively.

To explore the biological function of SH2B3 on TMZ therapy *in vivo*, we first established GBM xenograft tumors *via* injecting U87 cells. Fourteen days after GBM cells implantation, the mice were intraperitoneally injected with TMZ (60 mg/kg/day per mouse) or dimethyl sulfoxide (DMSO, 0.3%) twice a week for 6 weeks, and the therapeutic value of SH2B3 inhibition on TMZ therapy *in vivo* was evaluated. The mice receiving a combined treatment demonstrated a much smaller tumor volume than the control mice (Figures 5A–C). Additionally, the SH2B3 shRNA mice showed decreased levels of Ki67 positive cells as shown by IHC (Figures 5D,E). Overall, these data demonstrate that silencing of SH2B3 serves as a potential therapeutic target to augment the benefits of TMZ therapy.

### Silencing of SH2B3 Inhibits STAT3 Signaling

**FIGURE 6:**
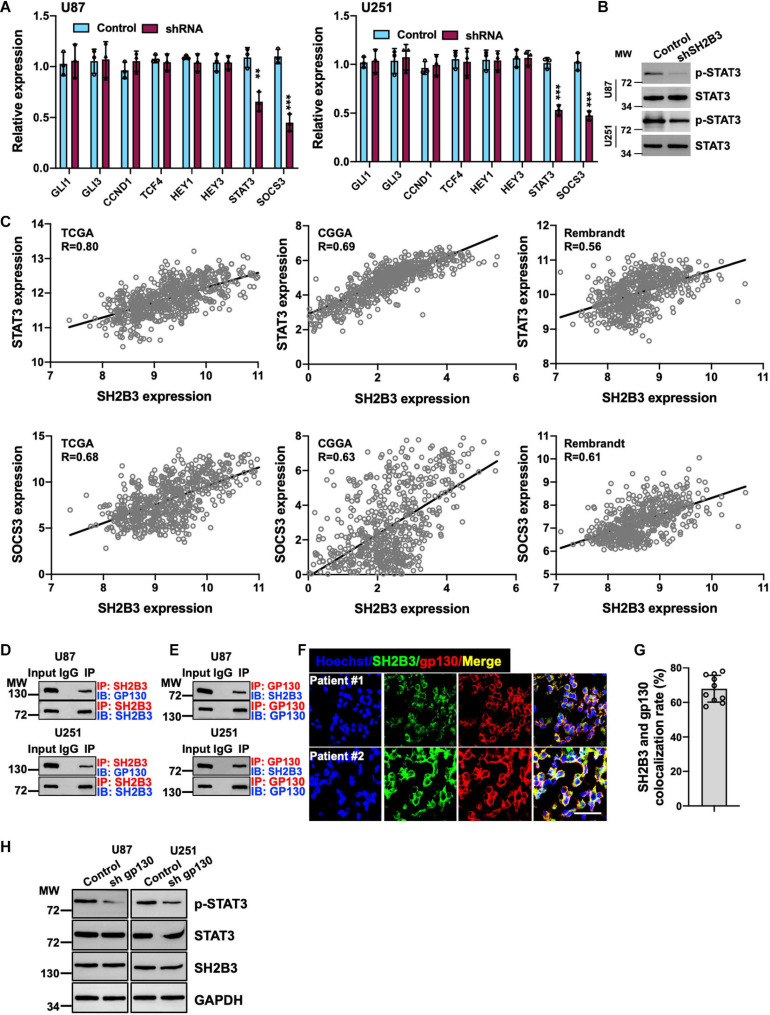
Silencing of SH2B3 inhibits STAT3 activation. **(A)** Target genes of STAT3 were dramatically decreased after silencing of SH2B3 in U87 (left panel) and U251 (right panel) cells. **(B)** Western blot of phosphorylation form of STAT3 (Y705). **(C)** SH2B3 mRNA expression was positively correlated with STAT3 target gene in indicated data sets. **(D)** SH2B3 physically binds to gp130. **(E)** Gp130 physically binds to SH2B3. **(F)** Representative images of co-immunofluorescence staining between SH2B3 and gp130 in patient-derived clinical specimens. Scale bar = 50 μm. **(G)** Quantification data for panel **(F)**. **(H)** Silencing of gp130 significantly attenuates p-STAT3 (Y705) expression. *, **, and *** indicate *p* < 0.05, *p* < 0.01, and *p* < 0.0001, respectively.

To further determine the underlying molecular mechanism by which SH2B3 regulates GSCs’ self-renewal, we assessed four major self-renewal signaling pathways, including Hedgehog (Das et al., 2013), Wnt/β-catenin (Nitta et al., 2013), Notch (Man et al., 2018), and STAT3 (Shi et al., 2017) signaling pathways, in which both of them are key regulators that have been well established to regulate GSCs’ self-renewal. We found that only STAT3 target genes, including STAT3 and SOCS3, were remarkably decreased in U87 and U251 SH2B3-deficient cells (Figure 6A). While we did not find any expression changes of Hedgehog, Wnt/β-catenin and Notch signaling pathways were affected following knockdown of SH2B3 (Figure 6A), suggesting that SH2B3 promotes GBM tumorigenesis by activating the STAT3 signaling pathways. In addition, the activated form of STAT3, p-STAT3 (Y705), was considerably decreased in SH2B3-deficient cells (Figure 6B). Consistently, we further examined the expression pattern of SH2B3 and those STAT3 target genes, and the results demonstrated that SH2B3 mRNA expression was positively correlated with those STAT3 downstream genes in TCGA, CGGA, and Rembrandt data sets (Figure 6C). Together, these data reveal that silencing of SH2B3 inhibits the STAT3 signaling pathway.

### SH2B3 Activates STAT3 Signaling by Physically Interacting With gp130

To further elucidate the underlying molecular mechanism by which SH2B3 activates STAT3 signaling, as gp130 is a well-established IL-6 receptor subunit (Shi et al., 2017), thus we hypothesized that SH2B3 may function as an adaptor protein and bind to gp130 to transduce IL-6/gp130 signaling. To test this hypothesis, we examined the potential binding probability between SH2B3 and gp130 using co-immunoprecipitation assay, and the results showed that SH2B3 physically and directly interacts with gp130 (Figure 6D). Furthermore, gp130 binds to SH2B3 as well (Figure 6E). This result was further confirmed by co-immunofluorescence staining. As shown in Figures 6F,G, SH2B3 co-localized with gp130. To further explore the underlying mechanisms by which SH2B3 activates STAT3 signaling, we stably knock down gp130 using lentivirus-mediated approaches in U87 and U251 cells. We found that p-STAT3 was considerably attenuated following silencing of gp130, which is consistent with a previous report (Shi et al., 2017; Figure 6H). On the other hand, we did not find any expression changes of SH2B3 following silencing of gp130 (Figure 6H), implying that SH2B3 functions as an adaptor protein to activate STAT3 signaling by transducing IL-6/gp130 signaling.

### SH2B3 Promotes GBM Progression in a STAT3-Dependent Manner

**FIGURE 7:**
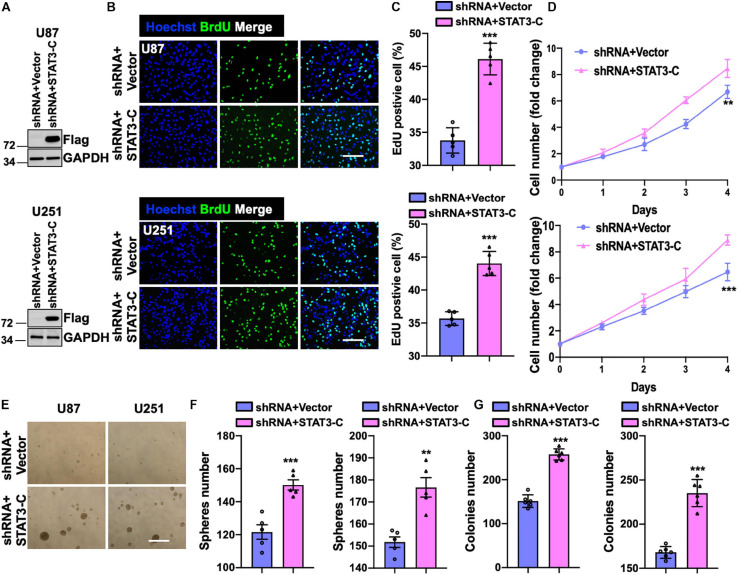
Forced expression of STAT3 restores overexpression of SH2B3. **(A)** Immunoblot of indicated protein in U87 (up panel) and U251 (below panel) SH2B3 silencing cells expressing with or without STAT3-C-Flag. **(B)** Immunofluorescence staining of BrdU in indicated GBM cells. Scale bar = 100 μm. **(C)** BrdU positive cells were quantified. **(D)** The cell proliferation rate of indicated GBM cells. **(E)** Representative images of tumor spheres derived from indicated GBM cells. Scale bar = 500 μm. **(F)** The number of tumor spheres was quantified. **(G)** The number of colonies was quantified. *, **, and *** indicate *p* < 0.05, *p* < 0.01, and *p* < 0.0001, respectively.

To examine whether the restored activity of STAT3 could compromise the suppressive effect of knocking down SH2B3, we introduced a Flag-tagged constitutively activated STAT3 (STAT3-C) into U251 and U87 cells expressing SH2B3 shRNA (Figure 7A). As expected, the forced expression of STAT3-C rescued U251 and U87 cells growth impaired by silencing of SH2B3 (Figures 7B–D). Tumor sphere formation (Figures 7E,F) and colony formation ability (Figure 7G) that impaired by disruption of SH2B3 were rescued by ectopia expression of STAT3-C, indicating that STAT3-C compromised the suppressive effect of silencing of SH2B3 on GSC maintenance. Collectively, these data demonstrate that SH2B3 suppressed GBM progression dependent on STAT3 signaling.

### Silencing of SH2B3 Suppresses Tumor Growth in Mouse Xenograft Model

**FIGURE 8:**
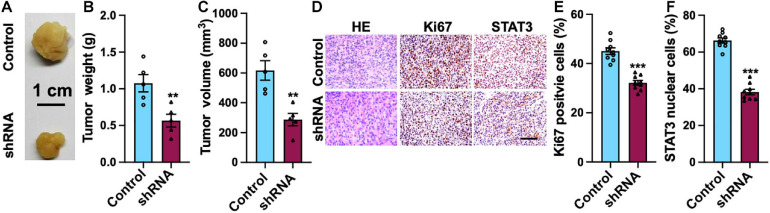
SH2B3 is essential for mouse xenograft tumors to grow *in vivo*. **(A)** Representative images of tumors derived from U87 control or SH2B3 shRNA cells. Scale bar = 1 cm. **(B)** Weight of indicated tumors. **(C)** The volume of indicated tumors. **(D)** Immunohistochemistry staining with tumors derived from U87 control or SH2B3 shRNA cells using indicated antibodies, and representative images were shown. Scale bar = 100 μm. **(E,F)** Quantification data for panel **(D)**. *, **, and *** indicate *p* < 0.05, *p* < 0.01, and *p* < 0.0001, respectively.

Mouse xenograft model was employed to study the functional role of SH2B3 in xenograft tumor growth. We observed that knockdown of SH2B3 significantly suppressed mouse xenograft tumor growth (Figures 8A–C). In addition, the tumors derived from mice were further performed by IHC staining, and we found that the nuclear localization of STAT3 from SH2B3 silencing cell-derived tumors was significantly accumulated in the cytoplasm and significantly reduced in Ki67 positive cells compared with control cell-derived tumors (Figures 8D–F). Collectively, these data reveal that SH2B3 inhibits mouse xenograft growth through suppressing STAT3 activation and nuclear localization.

## Discussion

Glioblastoma is the most common and aggressive brain tumor in adults. GSCs have been found to be enriched in GBMs after radiation and chemotherapy and contribute to therapeutic resistance and tumor re-initiation (Bao et al., 2006a; Chen et al., 2012). As STAT3 was frequently activated in GSCs and its aberrant activation is crucial for GSCs’ self-renewal and tumorigenic potential, targeting STAT3 signaling may potently suppress GSCs and have therapeutic potential. However, since STAT3 also plays essential roles in both normal cells and cancer cells in various biological processes (Yu et al., 2009; Guryanova et al., 2011), targeting STAT3 is not clinically achievable. Targeting upstream signaling of the STAT3 signaling pathway may provide new insight into addressing this problem. It is therefore urgent to identify new regulators of STAT3 signaling as promising clinical therapies.

Functions of SH2B3 were well recognized to negatively regulate Jak/STAT signaling and suppress hematopoietic cell growth (Seita et al., 2007; Simon et al., 2008; Balcerek et al., 2010; Taba-yashi et al., 2010). On the other hand, SH2B3 was reported to play paradoxical roles in solid tumor initiation and progression. On the contrary, previous studies show that SH2B3 promotes Jak/STAT signaling in myeloproliferative neoplasms (Oh et al., 2010) and promotes ovarian cancer (Ding et al., 2015), breast cancer (Lv et al., 2020), and anaplastic thyroid carcinoma (Zhong et al., 2020) progression. The biological function of SH2B3 in malignancy is affected by the context with tumor types. So far, little is known about the functions of SH2B3 in GSCs’ self-renewal and glioma tumorigenesis. Given that IDH1 mutation and epidermal growth factor receptor (EGFR) amplification are mutually exclusive events in GBM, high EGFR levels also correlate with patient survival. Since SH2B3 is overexpressed in IDH1 WT, we wonder if SH2B3 expression also correlates with EGFR levels. Regrettably, we did not find whether SH2B3 expression was significantly changed between EGFR WT and mutant group, implying that SH2B3 expression was not associated with the mutation status.

**FIGURE 9:**
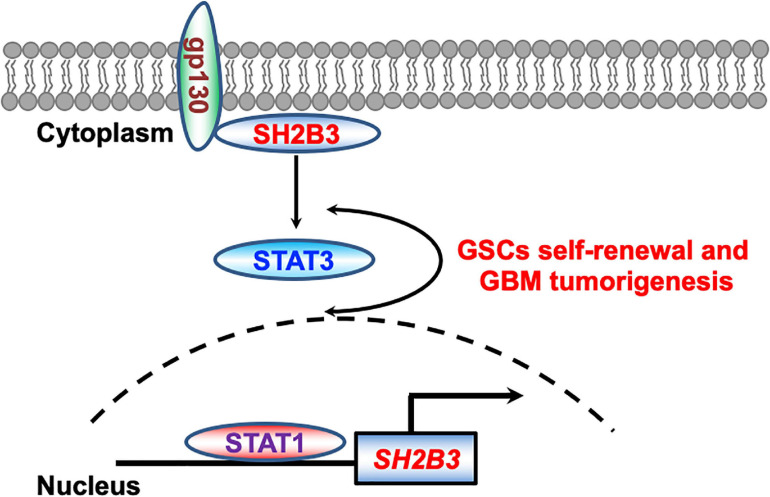
Working model. STAT1 directly binds to SH2B3 promoter and activates SH2B3 expression in the transcriptional level. SH2B3 functions as an adaptor protein to physically bind to gp130 and activates the STAT3 signaling pathways, thereby facilitating glioblastoma progression in turn.

Here, we report that SH2B3 was significantly highly expressed in GBM (WHO IV) compared with glioma (WHO II and III) and its high expression predicts worse clinical prognosis of glioma patients. In addition, we provide evidence that STAT1 directly binds to SH2B3 promoter and activates its expression in the transcriptional level (Figure 9), which in turn activates STAT3, thereby facilitating glioma progression. SH2B3 had been reported to regulate IL-7 receptor signaling in normal and malignant B-progenitor cells (Cheng et al., 2016). In the present study, we provide evidence that SH2B3 physically and directly interacts with gp130, which is a subunit of IL-6 receptor and functions as an adaptor protein to transduce IL-6/gp130 signaling, thereby activating STAT3 signaling and GBM tumorigenesis. Furthermore, we provide evidence that the STAT1/SH2B3/gp130/STAT3 signaling axis is required for GBM cell proliferation and GSCs’ self-renewal (Figure 9). Silencing of SH2B3 remarkably repressed GBM cell growth and tumor spheres as well as colony formation *in vitro* and xenograft tumor growth *in vivo* (Figure 9). However, whether the detailed molecular mechanisms of SH2B3 activate the STAT3 signaling pathway and promote GBM progression need our further study. Silencing of SH2B3 combined with TMZ therapy significantly augments TMZ suppression effect mediated by TMZ alone. Our study first demonstrated that SH2B3 was an upstream regulator of STAT3 and therapeutic drugs that directly target SH2B3 may be achievable for their ability to specifically decrease SH2B3/STAT3 signaling. However, up to date, there are no clinical drugs documented to inhibit SH2B3 expression. Thus, we will attempt to screen compounds that directly and specifically target SH2B3 to translate into clinical therapies for glioma patients.

SRC homology domain plays important roles in signaling transduction and multiple diseases initiation and development (Lin et al., 2020). The biological function of SH2B3 by which it promotes glioma initiation and progression may rely on binding with SRC homology domain. Whether the biological functions of SH2B3 depend on its SRC homology domain need further study. Our focus will be to further identify the detailed molecular mechanisms that SH2B3 transduces the IL-6/gp130/STAT3 signaling axis.

Taken together, our findings reveal a previously unknown signal by which SH2B3 mediates the STAT3 signaling pathways, thereby enhancing the oncogenic activity of STAT3 in human GBM. The newly established roles of SH2B3-driven tumorigenesis provide a putative GBM prognosis marker of SH2B3 in GBM and a new therapeutic target in the future.

## Data Availability Statement

The original contributions presented in the study are included in the article/supplementary material, further inquiries can be directed to the corresponding author/s.

## Ethics Statement

The animal study was reviewed and approved by the Application for Animal Experimental Ethical Inspection (KMMU 2020171).

## Author Contributions

XW designed the experiments, analyzed the data, and wrote the manuscript. SC performed almost all the experiments. JL performed the database analysis. YW, CS, and TY collected the GBM clinical samples and performed the IHC assay. All authors contributed to the article and approved the submitted version.

## Conflict of Interest

The authors declare that the research was conducted in the absence of any commercial or financial relationships that could be construed as a potential conflict of interest.

## Footnotes

1 http://bioinf.wehi.edu.au/software/elda/

2 https://www.genecards.org/

## References

[B1] Balcerek J. Jiang J. Bersenev A. Song Y. W. Wu C. Tong W. (2010). 14-3-3 Regulates the Lnk/JAK2 pathway in hematopoietic stem and progenitor cells. *Blood* 116 44–44. 10.1172/JCI59719 PMC3366400 22546852

[B2] Bao S. D. Wu Q. L. McLendon R. E. Hao Y. L. Shi Q. Hjelmeland A. B. (2006a). Glioma stem cells promote radioresistance by preferential activation of the DNA damage response. *Nature* 444 756–760. 10.1038/nature05236 17051156

[B3] Bao S. D. Wu Q. L. Sathornsumetee S. Hao Y. L. Li Z. Z. Hjelmeland A. B. (2006b). Stem cell-like glioma cells promote tumor angiogenesis through vascular endothelial growth factor. *Cancer Res.* 66 7843–7848. 10.1158/0008-5472.can-06-1010 16912155

[B4] Batchelor T. (2000). Temozolomide for malignant brain tumours. *Lancet* 355 1115–1116. 10.1016/s0140-6736(00)02055-9 10791369

[B5] Baysan M. Bozdag S. Cam M. C. Kotliarova S. Ahn S. Walling J. (2012). G-Cimp status prediction of glioblastoma samples using mRNA expression data. *PLoS One* 7:e47839. 10.1371/journal.pone.0047839 23139755 PMC3490960

[B6] Blass G. Mattson D. L. Staruschenko A. (2016). The function of SH2B3 (LNK) in the kidney. *Am. J. Physiol. Renal.* 311 F682–F685. 10.1152/ajprenal.00373.2016 PMC5142233 27440780

[B7] Brennan C. W. Verhaak R. G. W. McKenna A. Campos B. Noushmehr H. Salama S. R. (2014). The somatic genomic landscape of glioblastoma (Vol 155, pg 462, 2013). *Cell* 157 753–753. 10.1016/j.cell.2013.09.034 PMC3910500 24120142

[B8] Chen H. W. Aksoy I. Gonnot F. Osteil P. Aubry M. Hamela C. (2015). Reinforcement of STAT3 activity reprogrammes human embryonic stem cells to naive-like pluripotency. *Nat. Commun.* 6:7095. 25968054 10.1038/ncomms8095 PMC4479042

[B9] Chen J. Li Y. J. Yu T. S. McKay R. M. Burns D. K. Kernie S. G. (2012). A restricted cell population propagates glioblastoma growth after chemotherapy. *Nature* 488 522–526. 10.1038/nature11287 22854781 PMC3427400

[B10] Cheng Y. Chikwava K. Wu C. Zhang H. B. Bhagat A. Pei D. H. (2016). LNK/SH2B3 regulates IL-7 receptor signaling in normal and malignant B-progenitors. *J. Clin. Investigat.* 126 1267–1281. 10.1172/jci81468 26974155 PMC4811117

[B11] Das T. Chatterjee U. Ghosh S. N. Deb S. Saha S. K. Gulati P. (2013). An objective clustering of GBM patients to identify clinically relevant subgroup with Hedgehog pathway activity. *Cancer Res.* 73:5587.

[B12] Devalliere J. Charreau B. (2011). The adaptor Lnk (SH2B3): an emerging regulator in vascular cells and a link between immune and inflammatory signaling. *Biochem. Pharmacol.* 82 1391–1402. 10.1016/j.bcp.2011.06.023 21723852

[B13] Ding L. W. Sun Q. Y. Lin D. C. Chien W. Hattori N. Dong X. M. (2015). LNK (SH2B3): paradoxical effects in ovarian cancer. *Oncogene* 34 1463–1474. 10.1038/onc.2014.34 24704825 PMC4188804

[B14] Eckel-Passow J. E. Lachance D. H. Molinaro A. M. Walsh K. M. Decker P. A. Sicotte H. (2015). Glioma groups based on 1p/19q, IDH, and TERT promoter mutations in tumors. *New Engl. J. Med.* 372 2499–2508. 26061753 10.1056/NEJMoa1407279 PMC4489704

[B15] Gery S. Koeffler H. P. (2013). Role of the adaptor protein LNK in normal and malignant hematopoiesis. *Oncogene* 32 3111–3118. 10.1038/onc.2012.435 23045270

[B16] Guryanova O. A. Wu Q. L. Cheng L. Lathia J. D. Huang Z. Yang J. B. (2011). Nonreceptor tyrosine kinase BMX maintains self-renewal and tumorigenic potential of glioblastoma stem cells by activating STAT3. *Cancer Cell* 19 498–511. 10.1016/j.ccr.2011.03.004 21481791 PMC3076106

[B17] Hua L. Wang G. Wang Z. Fu J. Fang Z. Zhuang T. (2019). Activation of STAT1 by the FRK tyrosine kinase is associated with human glioma growth. *J. Neurooncol.* 143 35–47. 10.1007/s11060-019-03143-w 30993511

[B18] Kim S. S. Harford J. B. Moghe M. Rait A. Pirollo K. F. Chang E. H. (2018). Targeted nanocomplex carrying siRNA against MALAT1 sensitizes glioblastoma to temozolomide. *Nucleic Acids Res.* 46 1424–1440. 10.1093/nar/gkx1221 29202181 PMC5815062

[B19] Kohsaka S. Wang L. Yachi K. Mahabir R. Narita T. Itoh T. (2012). STAT3 inhibition overcomes temozolomide resistance in glioblastoma by downregulating MGMT expression. *Mol. Cancer Ther.* 11 1289–1299. 10.1158/1535-7163.mct-11-0801 22532597

[B20] Li H. W. Yuan X. L. Yan D. M. Li D. P. Guan F. X. Dong Y. (2017). Non-coding RNA MALAT1 decreases the sensitivity of resistant glioblastoma cell lines to temozolomide. *Cell. Physiol. Biochem.* 42 1192–1201. 10.1159/000478917 28668966

[B21] Liao Y. W. Shen L. F. Zhao H. T. Liu Q. Fu J. Guo Y. (2017). LncRNA CASC2 interacts with miR-181a to modulate glioma growth and resistance to TMZ through PTEN pathway. *J. Cell. Biochem.* 118 1889–1899. 10.1002/jcb.25910 28121023

[B22] Lin Z. Liu Z. Tan X. Li C. (2020). SH3GL3 functions as a potent tumor suppressor in lung cancer in a SH3 domain dependent manner. *Biochem. Biophys. Res. Commun.* 534 787–794. 10.1016/j.bbrc.2020.10.107 33168185

[B23] Lv J. X. Yu W. Zhang Y. A. Cao X. H. Han L. F. Hu H. L. (2020). LNK promotes the growth and metastasis of triple negative breast cancer via activating JAK/STAT3 and ERK1/2 pathway. *Cancer Cell Int.* 20:124. 32322171 10.1186/s12935-020-01197-9 PMC7160949

[B24] Man J. H. Yu X. J. Huang H. D. Zhou W. C. Xiang C. M. Huang H. H. (2018). Hypoxic induction of vasorin regulates Notch1 turnover to maintain glioma stem-like cells. *Cell Stem Cell* 22 104–118.e6. 29198941 10.1016/j.stem.2017.10.005 PMC5756127

[B25] Maslah N. Cassinat B. Verger E. Kiladjian J. J. Velazquez L. (2017). The role of LNK/SH2B3 genetic alterations in myeloproliferative neoplasms and other hematological disorders. *Leukemia* 31 1661–1670. 10.1038/leu.2017.139 28484264

[B26] Nitta R. Mitra S. Agarwal M. Bui T. Li G. (2013). Casein Kinase 2 alpha regulates Gbm cancer stem cell growth through the beta-catenin pathway. *Neuro. Oncol.* 15 211–211.

[B27] Noushmehr H. Weisenberger D. J. Diefes K. Phillips H. S. Pujara K. Berman B. P. (2010). Identification of a CpG Island methylator phenotype that defines a distinct subgroup of glioma. *Cancer Cell* 17 510–522. 20399149 10.1016/j.ccr.2010.03.017 PMC2872684

[B28] Oh S. T. Simonds E. F. Jones C. Hale M. B. Goltsev Y. Gibbs K. D. (2010). Mutation of the inhibitory adaptor protein LNK drives potentiated JAK-STAT signaling in patients with JAK2 V617F-negative myeloproliferative neoplasms. *Cancer Res.* 70:239. 10.1182/blood-2010-02-270108 PMC2924231 20404132

[B29] Ostrom Q. T. Cioffi G. Gittleman H. Patil N. Waite K. Kruchko C. (2019). CBTRUS statistical report: primary brain and other central nervous system tumors diagnosed in the United States in 2012-2016. *Neuro. Oncol.* 21 V1–V100. 31675094 10.1093/neuonc/noz150 PMC6823730

[B30] Perez-Garcia A. Ambesi-Impiombato A. Hadler M. Rigo I. LeDuc C. A. Kelly K. (2013). Genetic loss of SH2B3 in acute lymphoblastic leukemia. *Blood* 122 2425–2432. 10.1182/blood-2013-05-500850 23908464 PMC3790510

[B31] Raskin L. Guo Y. Du L. Clendenning M. Rosty C. Colon Cancer Family Registry (CCFR) et al (2017). Targeted sequencing of established and candidate colorectal cancer genes in the Colon Cancer Family Registry Cohort. *Oncotarget* 8 93450–93463. 10.18632/oncotarget.18596 29212164 PMC5706810

[B32] Rudemiller N. P. Lund H. Priestley J. R. Endres B. T. Prokop J. W. Jacob H. J. (2015). Mutation of SH2B3 (LNK), a genome-wide association study candidate for hypertension, attenuates Dahl salt-sensitive hypertension via inflammatory modulation. *Hypertension* 65 1111–1117. 10.1161/hypertensionaha.114.04736 25776069 PMC4412596

[B33] Seita J. Ema H. Ooehara J. Yamazaki S. Tadokoro Y. Yamasaki A. (2007). Lnk negatively regulates self-renewal of hematopoietic stem cells by modifying thrombopoietin-mediated signal transduction. *Proc. Natl. Acad. Sci. U.S.A.* 104 2349–2354. 10.1073/pnas.0606238104 17284614 PMC1892983

[B34] Shi Y. Zhou W. C. Cheng L. Chen C. Huang Z. Fang X. G. (2017). Tetraspanin CD9 stabilizes gp130 by preventing its ubiquitin-dependent lysosomal degradation to promote STAT3 activation in glioma stem cells. *Cell Death Different.* 24 167–180. 10.1038/cdd.2016.110 27740621 PMC5260495

[B35] Simon C. Dondi E. Chaix A. de Sepulveda P. Kubiseski T. J. Varin-Blank N. (2008). The Lnk adaptor protein negatively regulates specific SCF biological responses in primary hematopoietic cells. *Cytokine* 43 287–287. 10.1016/j.cyto.2008.07.264

[B36] Sinclair P. B. Ryan S. Bashton M. Hollern S. Hanna R. Case M. (2019). SH2B3 inactivation through CN-LOH 12q is uniquely associated with B-cell precursor ALL with iAMP21 or other chromosome 21 gain. *Leukemia* 33 1881–1894. 10.1038/s41375-019-0412-1 30816328 PMC6756024

[B37] Stupp R. Mason W. P. van den Bent M. J. Weller M. Fisher B. Taphoorn M. J. B. (2005). Radiotherapy plus concomitant and adjuvant temozolomide for glioblastoma. *New Engl. J. Med.* 352 987–996. 15758009 10.1056/NEJMoa043330

[B38] Taba-yashi T. Gery S. Koren-Michowitz M. Koeffler H. P. (2010). Adaptor protein Lnk negatively regulates Bcr-Abl-induced cell proliferation through inhibition of the Stat5 signaling pathway. *Blood* 116 1394–1394.20813906

[B39] Turcan S. Rohle D. Goenka A. Walsh L. A. Fang F. Yilmaz E. (2012). IDH1 mutation is sufficient to establish the glioma hypermethylator phenotype. *Nature* 483 479–U137. 22343889 10.1038/nature10866 PMC3351699

[B40] Van Meir E. G. Hadjipanayis C. G. Norden A. D. Shu H. K. Wen P. Y. Olson J. J. (2010). Exciting new advances in neuro-oncology the avenue to a cure for malignant glioma. *CA Cancer J. Clin.* 60 166–193. 10.3322/caac.20069 20445000 PMC2888474

[B41] Wang W. Tang Y. Wang Y. Tascau L. Balcerek J. Tong W. (2016). LNK/SH2B3 Loss of function promotes atherosclerosis and Thrombosis. *Circ. Res.* 119 e91–e103. 27430239 10.1161/CIRCRESAHA.116.308955 PMC5016083

[B42] Yu H. Pardoll D. Jove R. (2009). STATs in cancer inflammation and immunity: a leading role for STAT3. *Nat. Rev. Cancer* 9 798–809. 10.1038/nrc2734 19851315 PMC4856025

[B43] Yung W. K. A. Lieberman F. S. Wen P. Robin I. Gilbert M. Chang S. (2005). Combination of temozolomide (TMZ) and irinotecan (CPT-11) showed enhanced activity for recurrent malignant gliomas: a north american brain tumor consortium (NABTC) phase II study. *J. Clin. Oncol.* 23:119s.

[B44] Zhao L. Li X. Su J. Wang Gong F. Lu J. Wei Y. (2020). STAT1 determines aggressiveness of glioblastoma both in vivo and in vitro through wnt/beta-catenin signalling pathway. *Cell Biochem. Funct.* 38 630–641. 10.1002/cbf.3518 32390230

[B45] Zhong Z. M. Chen X. Qi X. Wang X. M. Li C. Y. Qin R. J. (2020). Adaptor protein LNK promotes anaplastic thyroid carcinoma cell growth via 14-3-3 epsilon/gamma binding. *Cancer Cell Int.* 20:11. 31938019 10.1186/s12935-019-1090-9 PMC6953139

